# Addressing Multipath Signal Corruption in Microwave Tomography and the Influence on System Design and Algorithm Development

**DOI:** 10.32474/OAJBEB.2018.01.000102

**Published:** 2018-02-05

**Authors:** Paul M Meaney, Keith D Paulsen

**Affiliations:** 1 Thayer School of Engineering, Dartmouth College, USA; 2 Electrical Engineering Department, Chalmers University of Technology, Sweden

## Abstract

In developing a microwave tomography system, we started by examining the fundamental signal measurement challenges-i.e., how to interrogate the target while suppressing unwanted multi-path signals. Beginning with a lossy coupling bath to suppress unwanted surface waves, we have developed a robust and reliable system that is both simple and low profile. However, beyond the basic measurement configuration, the lossy coupling medium concept has also informed our choice of array antenna and imaging algorithms. The synergism of these concepts has produced a novel concept which is embodied in a system that has been successfully translated to the clinic.

## Introduction

While there are often numerous ways to approach scientific challenges, some are often more productive than others. In the area of microwave tomography, the vast majority of efforts have started with and focused on developing algorithms to deal with the inherent nonlinearity of the problem [1,2]. By itself, these nonlinear problems are very challenging and the genesis for numerous reports, books and conferences. Conversely, our motivation began with the simple question of assessing whether we could gather suitable measurement data and what were the fundamental challenges. Following this line of enquiry, we quickly realized, as was well known in related microwave applications, that multi-path signal corruption was the single greatest factor in confounding our measurement systems [3]. As we became aware of over time, multi-path signals come in a variety of forms including reflections off of illumination chamber walls, surface waves along support structures and feed lines and even as cross-channel leakage within the system electronics [4,5]. It is not a matter of eliminating them, but attenuating them to a sufficient level that their impact on the desired signals is tolerable.

At an early stage, we became aware that surface waves were particularly problematic in near field measurement systems. These waves are capable of traveling large distances with only minimal degradation and can readily re-couple through an alternative propagation path and corrupt the desired signals [6,7]. Our earliest solutions were to utilize lossy coupling baths such as saline and mixtures of glycerin and water to attenuate the signals [8,9]. In addition, it was important to maintain long enough feed line lengths and keep the tank boundaries sufficiently far from the antennas and field of view [4,5]. While inconvenient because of the practical challenges involved with working with liquids, it became a useful benchmark for experimenting with different configurations. An equally daunting problem involves the fact that the desired signals are also substantially attenuated by the lossy medium, to the extreme that we needed to develop custom measurement equipment to measure the signals down to abnormally low power levels [10]. Interestingly, the levels required are essentially out of the range of conventional test and measurement equipment, but not impractically impossible given theoretical limits [11]. The challenge becomes a trade-off between a nearly impossible problem for most approaches- i.e., suppressing or accounting for multi-path signals-versus developing a customized, multi-channel, high dynamic range measurement system- a dauntingly challenging problem, but eminently doable.

The insights gleaned from this line of investigation have informed a range of system design decisions and shaped the overall framework of our system. In terms of antenna design, conventional wisdom encourages one to design larger antennas that both operate over broader bandwidths and include higher gain levels to propagate across larger distances and through lossier material [12]. Conversely, we opted for extremely simple monopole antennas because, while they essentially have no gain, their small size allows them to be packed closer to the target which more than compensates for the lack of gain associated with their larger and more directive counterparts [12]. For the imaging algorithm, we rely on classic engineering observations and intuition in dealing with signal levels that span many orders of magnitude. Microwave engineers have long displayed signals in terms of their log magnitude and phase as a way to derive critical insights into the impacts of circuit, antenna and system designs [13].

**Figure 1:**
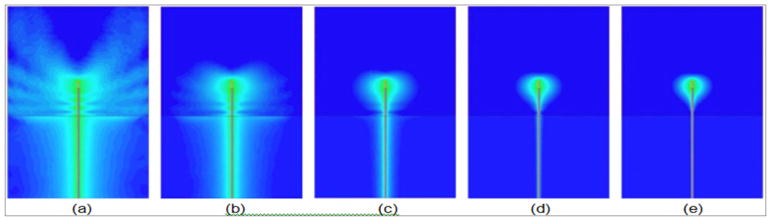


In fact, the ubiquitous default measurement display on virtually all commercial test and measurement equipment is in terms of decibels (the most widely used logarithmic-based scale) and degrees. The rationale is particularly prescient when measuring signals for antennas operating in a lossy bath. We exploit algorithms developed for competing imaging modalities that equivalently operate with highly attenuating media such as optical coherence tomography [14,15]. Only after use for a number of years did we discover that the mathematics underpinning this concept were developed and validated many decades ago [16]. In fact, these alternative means of framing the algorithmic challenges have led to approaches that no longer require a priori information for reconstruction convergence which is one of the more vexing problems for microwave tomography and we can now recover quality images in a fraction of the time and with fewer computational resources than competing concepts [17]. Figure 1: Simulated radiation patterns for a monopole antenna submerged in a coupling liquid above a slab of Plexiglas for different conductivity values: (a) 0.0, (b) 0.2, (c) 0.5, (d) 0.9, and (e) 1.2 S/m, respectively.

In the following sections, we more closely describe the fundamental challenges in developing microwave tomography in the context of each of the practical issues outlined above. Ultimately, the synergism of novel solutions to each of these issues have contributed to an overall concept that has led to deployment of actual imaging technology that has now been used in various medical scenarios [18,19]. While the different solutions may appear counterintuitive in an isolated context and contrary to alternative approaches in the microwave imaging world, when viewed together, they comprise a range of fundamental solutions to difficult challenges which have been key to clinical success [20].

## Design Trade-Offs

### Multi-Path Suppression Via Lossy Coupling Bath

Scientists have been aware of the surface wave phenomenon for many decades [21]. While researchers are currently looking to exploit this behavior [22], for many applications the goal is to suppress them [6,7]. One of the more perplexing challenges with surface waves is that they are the same frequency as the desired signal and can appear as if they are the desired scattering from an object in the field of view, similarly to how the human body’s defense mechanism has trouble distinguishing cancers from normal tissue. Figure 1 shows a series of simulations of a monopole antenna radiating into a dielectric space above a solid slab of Plexiglas (ε_r_ = 2.2, σ = 0.0S/m). The permittivity of the coupling liquid was ε_r_= 20.0 and the conductivity were artificially varied from 0.0 to 1.2S/m, the latter being typical for an 80% concentration of glycerin in water. The length of the coaxial feed line above the Plexiglas was 14 cm and the length of the monopole antenna was 3.5 cm. There are several important features to note from these results. For the lowest conductivity case, the radiation pattern of the antenna is quite erratic, propagating in multiple directions.

There is also a significant coaxial mode traveling down the outside of the cable and right on into the Plexiglas. At the interface between the liquid and Plexiglas, it also excites a planar mode which travels along the interface with only minor attenuation as it moves away from the coax. As the conductivity increases to 1.2S/m, the aberrant beam pattern of the monopole antenna diminishes but the coaxial and planar surface wave modes are still prevalent. Interestingly, for the planar mode, the signal strength is decidedly stronger on the Plexiglas side of the interface indicating that the waves propagate preferentially on the lower loss side of a boundary.

As the attenuation increases further, the horizontal planar mode disappears and eventually the entire coaxial mode disappears- essentially the coaxial mode is completely eliminated before it has an opportunity to couple to the coax in the Plexiglas. In the un-attenuated or under-attenuated scenarios, these coaxial and planar modes are exactly the propagation paths that generate the corrupting multi-path signals. In situations such as a near-field, multi-antenna array, there is simply insufficient distance between elements to keep the unwanted signals from interfering with the desired ones. Simple signal analysis shows that when the undesired signals are 25dB below that of the desired signals, the maximum amplitude and phase errors can be as large as 0.1 dB and 3.2 degrees. These can increase to as much as 0.4 dB and 17.5 degrees for a multi-path signal that is only-10 dB below the desired one. These types of errors could dramatically impact the overall image quality. While the lossy bath is a real, albeit inconvenient solution, there is a conspicuous lack of viable alternatives in the near field scenario. The multipath challenge has proven to be the most challenging aspect in developing an actual system.

**Figure 2:**
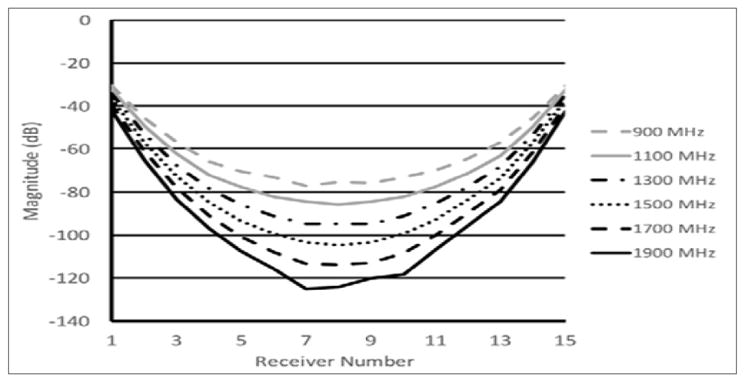
Plot of the attenuation for signals radiated from a single monopole antenna Into 70% glycerin:water bath and received at the complementary 15 array antennas arranged on a 15.2 cm diameter circle.

One major drawback for using this approach is that the desired signal strength is dramatically reduced at the receiver antennas. Fig. 2 shows plots of the S21 (transmission) measurements for a single monopole antenna radiating into a homogeneous, 70% glycerin bath and being received by the complementary antennas positioned on a 15.2 cm diameter circle (measurements were acquired utilizing a Rohde & Schwarz ZNBT8 16-channel vector network analyzer). As can be seen, the attenuation at the furthest antennas can easily exceed 120 dB which exceeds the capability of most commercial VNA’s. In fact, the primary limitation is not necessarily physical, because the noise floor can theoretically be driven arbitrarily low by dramatically increasing the IF measurement bandwidth [11].

For most devices, the limitation is most likely related to meeting the needs of the majority of customers who would rarely require such an expanded dynamic range. While the R&S VNA is a useful option, in prior efforts we chose to fabricate custom equipment to meet these specifications. It is worth noting that there is a wide misconception on how to configure a measurement system that has essentially equivalent consequences. The most typical measurement configuration is to utilize a single, two-channel VNA in conjunction with a 2 x 16 channel multiplexer [23,24]. The challenge is that most switching networks rarely have isolation greater than 80 dB. This would effectively reduce a VNA with a 100dB dynamic range to one with fewer than 80 dB. If the requirement is greater than 100dB, the switch matrix solution can cause even more problems.

### Antenna Choice

In conventional applications, mainly involving propagation in air, monopole antennas are extremely narrow band, inefficient and excite unwanted surface waves [6,7]. However, when immersed in a lossy coupling medium, the antennas effectively become resistively loaded and are reasonably well matched over a broad frequency range [25]. Unfortunately, the resistive loading also includes the unwanted feature of significantly reducing the overall efficiency. From a system design perspective, the primary goal is to measure a signal with sufficient SNR so that it can be used in an imaging algorithm. A classic means of doing this is to select an antenna with reasonable gain for both transmitter and receiver. However, this invariably implies utilizing a larger antenna-the gain is typically proportional to its size [12].

In a near field setting, this is problematic because the trade-off then becomes the number of antennas versus the surrounding array diameter. Decreasing the number of antennas would induce unintended consequences such as reduced image quality because of the lower number of measurements [26]. Increasing the array diameter is also suboptimal because of the added signal loss between antennas. As was illustrated above in Figure 2, the signal attenuation in these lossy baths is dominated by the plane wave attenuation through the conductive medium. By selecting the low profile monopole antennas and placing them close to the target, the plane wave attenuation is dramatically reduced by virtue of the shorter propagation paths with only minimal degradation in gain since there would be practical limits on the antenna sizes. A further and not insignificant benefit is that these antennas have virtually no mutual coupling effects even when packed quite closely [27].

Beyond this fundamental trade-off, the monopole presents further opportunities in the context of microwave imaging. For instance, researchers have speculated on ways of exploiting multi-frequency and even time-domain imaging techniques [28–30]. In these instances, it is critical to utilize broadband antennas. Our current model operates from roughly 500MHz to 3GHz with nominally 10 dB return loss or better across the band which is quite broad within the context of available alternatives [25]. Interestingly, as will be discussed below, for the log transform-based algorithm, the signal phases need to be unwrapped. We have pioneered a novel strategy of exploiting the multi-frequency information for the unwrapping [20].

As we progress towards lower cost and even semi-portable imaging system designs, we are continually finding new opportunities utilizing the monopole antennas. Recently we have begun using the discrete dipole approximation (DDA) as a means for efficiently computing the forward solution part of the reconstruction algorithm [31]. This has produced a nearly order of magnitude reduction in computation time. However, to take advantage of this concept, it is critical that the field propagation occur in a low scattering environment - preferably no metal. Because of the low profile of the monopole antennas and the lossy coupling bath, the presence of the non-active antennas (i.e. the remainder of the surrounding array) has very little impact on the field distribution and the low scattering criteria is met. These advantages and more obvious ones including the reduced illumination tank size have been crucial in translating this technology into the clinic.

### Reconstruction Algorithm

**Figure 3:**
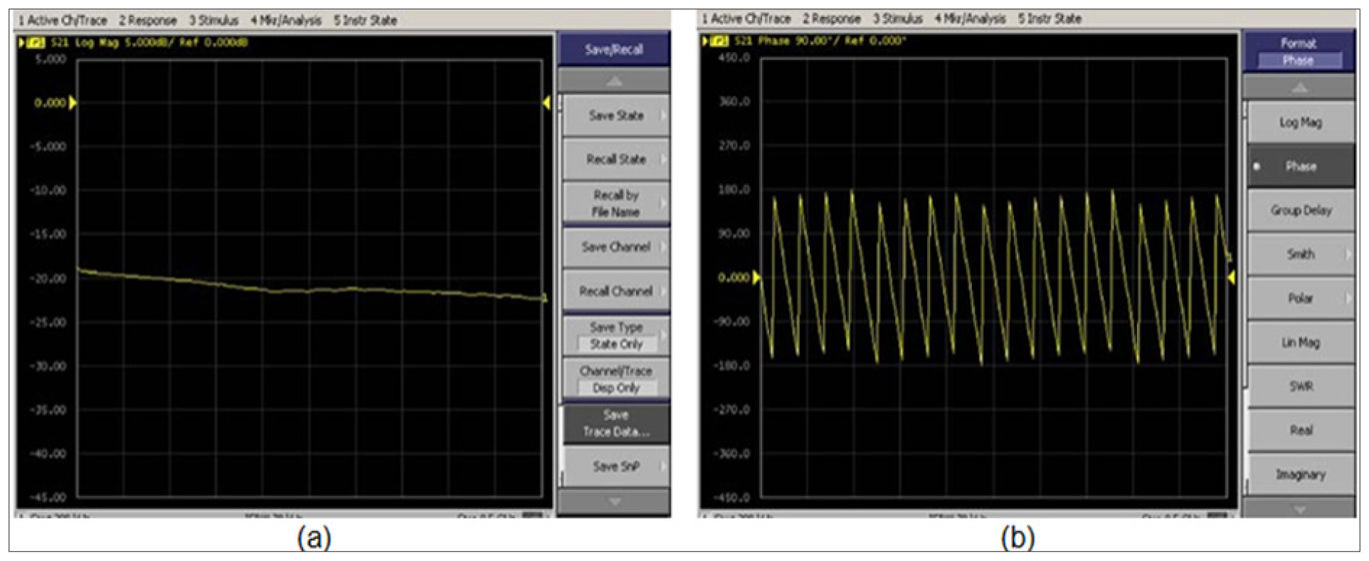


Researchers and engineers have long known that displaying electric field values in terms of decibels and phase provides a more intuitive appreciation of the signals versus viewing them in terms of the real and imaginary parts of the complex signal. In fact, the log magnitude and phase are simply the natural results of the log transformation of the complex signal [32]. For instance, in radar applications, the phase is generally directly related to distance, and with a modest amount of phase unwrapping can be used for range finding [11]. In fact, for most test and measurement equipment, the default display settings are the log magnitude and phase (Figure 3). This insight translates directly into the imaging world. In x-ray computed tomography (CT), while the reconstruction algorithm is linear and the data is purely real, it only operates properly after the measurement data has been logarithmically transformed [33]. Figure 3: Displays of an Agilent E5071B vector network analyzer measuring the transmission loss of a 20 dB coaxial attenuator and length of cable in both the (a) log magnitude and (b) phase modes.

More recently, the log transform has been widely used in optical coherence tomography (OCT) [14]. A large part of the advantage in this situation is that the amplitudes of the signals span many orders of magnitude. As alluded to above, one of the main challenges with the log transform is the question of how to handle the phase and possible wrapping. This is generally not an issue for OCT since they use transport equations for the light propagation modeling and artificially introduce the phase by way of a low frequency modulation-for breast imaging they typically use 100 MHz [34–36]. In this situation, the wavelength is sufficiently long that the phase never exceeds + /− π.

Exploiting the log transformation in microwave tomography presents important opportunities and challenges. In the context of measuring signals many orders of magnitude lower than the initially broadcast fields, the log scale is ideal for distinguishing signals. However, because scattering objects in the field of view can be relatively high contrast with the background and often on the order of a wavelength or more size-wise, the phases can easily wrap over the + /−π boundaries. Unwrapping the phases is a critical step for the reconstruction algorithm because it is crucial that the measured and computed phases (those calculated at each iteration) be on the same Riemann sheet [32]. For this challenge, we rely heavily on insights from within the microwave community but also from external technologies.

Phase unwrapping is critical for certain radar approaches used in range finding [11,37]. Simply viewing the phase wrapping on the displays of a vector network analyzer, the unwrapping process is relatively obvious with respect to phase shifts as a function of frequency [13]. Alternatively, there is a broad variety of literature in the magnetic resonance (MR) imaging world related directly to phase unwrapping [38,39]. While a natural approach might be to unwrap the phases as a function of spatial position, we have found that this is often complicated and computationally intensive [40]. For our application, we treat the measured and computed values differently. Because our antennas operate over a very broad bandwidth, it is possible to unwrap the measured phases as a function of frequency similarly to that just described for the VNA display [20]. This would not be feasible for the computed phases since it would require calculating entire phase distributions at multiple frequencies.

However, because we deliberately keep the iteration step size short between reconstruction iterations, the computed phase changes at each iteration for each receiver antenna rarely exceed π/10. Because of this, it is possible to conveniently unwrap the phases at each receiver antenna as a function of iteration with only minimal extra computational costs [20]. One of the more intriguing and powerful consequences of developing this approach is that the algorithm no longer requires a priori information and does not converge to local minima or unwanted solutions [20]. Much literature has been expended in finding solutions to this problem [41,42]. Our working hypothesis is that when alternative approaches reconstruct images utilizing the complex representation of the fields, the field values are automatically mapped into the single Riemann sheet spanning-π to +π [43]. For a significant portion of the data, this can mean a loss of information.

The application of a priori information can sometimes mitigate this problem, but it is essentially moving the computed field values to the same Riemann sheet as the measured data. For the most part, a priori information remains more of a mathematical construct than something that could be implemented in an efficient manner in the real world. Multiple groups have implemented frequency hopping approaches to some benefit [44,45]. Similarly to the a priori information approach, this technique produces an initial image at a low frequency which is unlikely to exhibit phase wrapping in the measurement data because of the longer wavelengths. This image is subsequently used as the starting guess for the next higher frequency. Even though the data at this higher frequency might show wrapping effects, the improved initial image estimate succeeds because it forces the computed and measured phase values onto the same Riemann sheet. This process is repeated until an image is reconstructed at the highest possible frequency. This elaborate and time-consuming process can be effective, but is ultimately unnec essary in light of the unwrapping procedure previously described.

**Figure 4:**
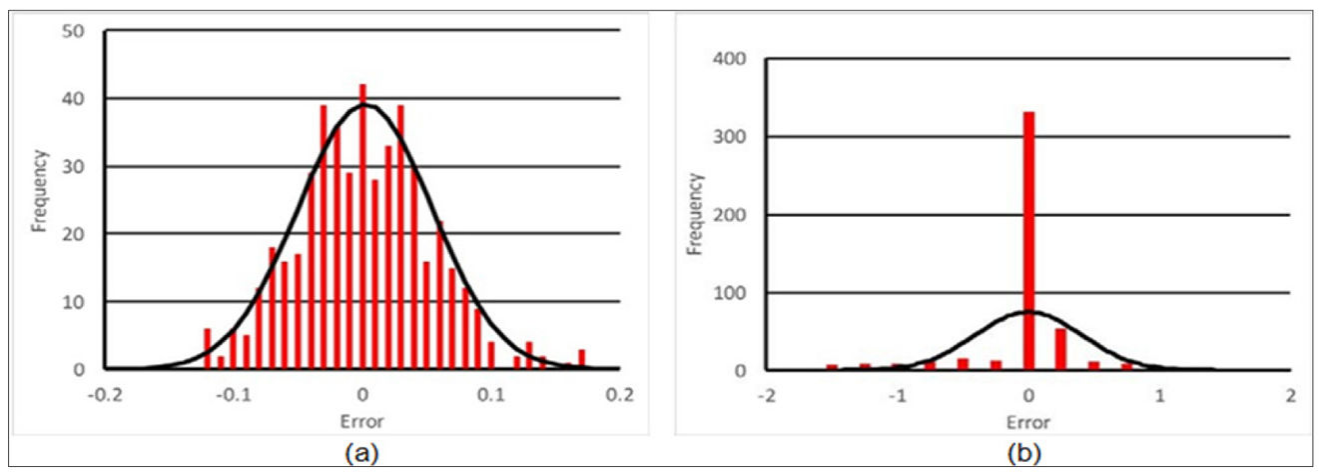
Histograms of the residual field error for phantom experiment image reconstructions (a) with the log transformation, and (b) without.

More recently, we have also discovered that the log transformation is actually well grounded in fundament analyses for general parameter estimation problems. Seminal work by Box and Cox described and assessed a broad range of transformations for parameter estimation, especially when the estimations exhibit different forms of hetero-scedasticity [16,46]. One of the major underpinnings of least squares approaches such as our Gauss-Newton technique is that the error between the measured and computed values have zero mean and are normally distributed. In an analysis similar to that performed in Meaney [46], Figures 4a & 4b show histograms for a different phantom imaging experiments with both the log transformation and without it, along with a best fitting normal distribution. These results are typical of what we encounter for both phantom experiments and clinical examinations and confirm that the log transformation provides more efficient minimization criteria for the reconstructions.

## Conclusion

In focusing on the more practical issues surrounding developing a microwave tomography system, we have solved critical challenges. However, the solutions can be viewed as counterintuitive and unconventional in light of competing solutions. These solutions include using a lossy coupling medium, monopole antennas and a log transformed reconstruction algorithm. Further examination of these approaches has led to important new innovations that now make microwave tomography a viable modality, especially as we address lower cost and more portable configurations for developing world applications.
